# Handling of the Venereal Problem in United States Army in the Present Crisis

**Published:** 1919

**Authors:** 


**Handling of the Venereal Problem in United States Army in the Present Crisis.** By W. A. Pusey. *The Journal of the American Medical Association*, September 28, 1918.

The author speaks of the difficulty encountered in handling venereal diseases owing to the social, personal and moral factors involved, and proposes a program in which extreme measures have been carefully avoided.

*Methods of Prevention.* (1) Social measures to diminish sexual temptation.

(2) Education in regard to venereal diseases.

(3) Prophylactic measures against venereal diseases.

(4) Medical care.

*Social Measures.* Social measures divide themselves into two sorts of activities :

(1) The repression of prostitution and liquor traffic, calling for co-operation between federal and local authorities. In accordance with a decision of the Secretary of War, the liquor traffic is controlled by the federal authorities within 5 miles around each cantonment. The local authories have done their best, and a marked improvement has been noted, thanks also to the help given by such associations as the American Social Hygiene Association.

(2) The provision of proper surroundings and of opportunities for recreation and diversion.

Here the author records the work of the Y. W. C. A., the Knights of Columbus, and the Y. M. C. A., as well as the aid brought by the civil population.

*Education.* The author speaks of the work done in the education of men, women, boys and young girls : lectures, distribution of pamphlets, etc., by the General Federation of Women's Clubs, Y. W. C. A., the Women's Temperance Union, the Young Women's Patriotic League, and other organizations. In the army, the same type of work has been carried out under the guidance of the War Department Commission on Training Camp Activities, and the Social Hygiene Section of the Surgeon-General's Office.

*Segregation of Prostitutes.* The segregation of prostitutes and the regulation of prostitution in the continental sense; i. e., the examination of prostitutes, their certification, and their toleration in definite districts, have not been regarded as advantageous and have not been included in this program.

*Medical Program.* The first part of this program is prophylaxis to prevent infection after exposure. Venereal prophylaxis is a part of the duty of every infirmary in care of the health of a unit. The number of cases of venereal infection that develop after prophylaxis is extremely small. Excellent work has been done in that respect in the army. Among the measures responsible for the reduction of venereal disease the two following must be noted; loss of pay which disability from venereal disease entails, and the fortnightly physical inspections.

*Medical Care of Venereal Diseases.* An effort has been made to standardize as far as possible the handling of venereal diseases. Acute cases must be hospitalized; every hospital should be provided with a complete laboratory in which Wassermann tests are to be made; and the treatment of venereal cases must be conducted along one definite line. Laboratories already exist in various military hospitals, and the authorities have endeavored to increase efficiency in venereal diseases by establishing special training schools.

*Quality of Treatment Venereal Patients are Receiving.* As a whole, venereal diseases receive excellent treatment. Acute cases of syphilis and chronic gonorrhea receive better attention at the base hospital than in the division.

*Results.* The venereal situation in the U. S. Army has been excellent for several years. The Army record for venereal diseases for several years past is as follows : Previous to 1898 the venereal rate averaged about 80 to 85 per 1000. With the mobilization of new troops in the Spanish-American War in 1898, the rate suddenly doubled, going to 160. It varied between 160 and 180 until 1911. From a rate of 164 in 1911, it fell to 116 in 1912, and 86 in 1913. Since 1913 the rate has remained under 90, except in 1916 when it went to 91.4. With the second week of mobilization the venereal rate for the National Army went up to 367, and the National Guard showed a rate of 150. In spite of the sudden increase caused by mobilization, the rate of the whole U. S. Army for 1917 was 88, as compared with 91. 3 for 1916. The figures since December 21 are these : 75, 57, 74, 58, 52, 55, 44, 48, i. e., running nearly to one-half of what might have been expected as a reasonable showing when the war began.

